# Editorial: Gastrointestinal tract barrier damage in health and in inflammatory diseases

**DOI:** 10.3389/fphys.2022.1038314

**Published:** 2022-09-23

**Authors:** Daniele Maria-Ferreira, Elizabeth Soares Fernandes, Carolina Silva Schiebel, Suzany Hellen da Silva Soczek, Marcelo Biondaro Gois, Lívia Sousa Ribeiro, Lucas Antonio Duarte Nicolau, Luiz Fernando Lopes Soares Teixeira

**Affiliations:** ^1^ Instituto de Pesquisa Pelé Pequeno Príncipe, Faculdades Pequeno Príncipe, Curitiba, Brazil; ^2^ Programa de Pós-graduação em Biotecnologia Aplicada à Saúde da Criança e do Adolescente, Faculdades Pequeno Príncipe, Curitiba, Brazil; ^3^ Instituto de Ciências da Saúde, Universidade Federal da Bahia and Centro de Ciências da Saúde, Universidade Federal do Recôncavo da Bahia, Cruz das Almas, Brazil; ^4^ Programa de Pós-Graduação em Biotecnologia, Ppgbiotec, Universidade Federal do Delta do Parnaíba, UFDPar, Parnaíba, Brazil

**Keywords:** intestinal mucosal barrier, intestinal damage, intestinal inflammation, intestinal diseases, inflammatory bowel diseases

The semipermeable mucosal surfaces of the gastrointestinal tract are responsible for nutrient absorption and waste secretion (Soderholm and Pedicord, 2019), and importantly, act as protective barriers between hostile external agents and the host internal environments to promote homeostasis (Turner, 2009). Recent advances have described numerous new information on how the intestinal mucosal barrier is regulated in light of normal physiological conditions and harmful circumstances. In our Research Topic, we received articles that contribute to the advancement of the knowledge already acquired. Six articles were accepted for publication.

One of the published articles is a literature review. In a very well-written, fluid, and organized review, Yang and colleagues provided an update on the existing knowledge of the role of mechanosensitive ion channels and intestinal diseases. Yang et al. described the importance of mechanical sensitization for the normal function of the gastrointestinal tract, and how abnormalities may be associated with the development of intestinal disorders. In addition to reviewing the functions of each mechanosensitive ion channel (Piezo channels; voltage-gated ion channels; transient receptor potential channels (TRP); two-pore domain potassium channels (K2o); large-conductance Ca_2_^+^-activated K+ channels (BKCa), and the cystic fibrosis transmembrane conductance regulator (CFTR), the authors describe their expression sites and definite roles in the gastrointestinal tract. In specific topics of the review, the authors described how mechanosensitive ion channels can alter transmembrane ion currents in response to stimuli, and how these alterations can be associated with gastrointestinal disorders. The authors highlighted that mechanosensitive ion channels may represent interesting targets for the treatment of gastrointestinal diseases.

Five original articles were published on this Research Topic. The first by Droessler and colleagues demonstrated through the Ussing chamber technique, the effects of tumor necrosis factor on the epithelium of porcine Peyer’s patches. The transepithelial electrical resistance of epithelial tissue specimens of Peyer’s patches and the surrounding villus epithelium incubated with TNF, the expression of epithelial tight junction proteins, and tumor necrosis factor receptors 1 and 2 were explored. A reduction in transepithelial electrical resistance was observed in Peyer’s patch tissue samples, but not in villus epithelium. The expressions of claudin 1 and 4 were decreased, while that of claudin 2 was enhanced. Increased TNF receptor 2 expression was observed in the Peyer’s patches. The authors demonstrated that TNF alters the normal function of the epithelial barrier of porcine Peyer’s patches without altering the villus epithelium, highlighting the significant functional and molecular differences between these two structures and their roles in the primary immunological defense of the mucosa. These results may contribute to the development of new therapeutic approaches for inflammatory bowel problems.

Busch and colleagues explored the role of the NLRP3 inflammasome pathway in acute intestinal inflammation. To carry out this study, the authors used a triple culture model (Caco-2/HT29-MTX-E12/THP-1) and intestinal tissue explants from wild-type and Nlrp3^−/−^ mice. Through well-designed experiments and sophisticated techniques, the authors showed that intestinal inflammation induced by NLRP3 inflammasome activators PS-NH2, DQ12, LPS, or a combination of LPS and IFN-γ impairs the intestinal barrier, by altering the expression of cytokines and mucins. Gene deletion of CASP1 and NLRP3 in THP 1 cells attenuated this alteration, especially in CASP1 knockouts. Furthermore, the reduction of mucus-secreting cells was NLRP3/caspase-1-dependent, and inflammation was observed in ileal tissue explants from WT, but not Nlrp3^−/−^ mice. The study provides important information on the adverse and pro-inflammatory roles of NLRP3 activation in macrophages.

In another study, Jimenez et al. used Tspo−/− mice to explore the role of translocator protein (TSPO) in DSS-induced ulcerative colitis. The results found allowed us to observe that the experimental animals (with ulcerative colitis) were seriously ill compared to the control animals (Tspofl/fl). Epithelial integrity loss, extensive tissue inflammation, and excessive mast cell activation were observed in these animals. Using molecular techniques to evaluate mRNA gene expression, an increase in highly sensitive and specific inflammatory markers dx2, mast cell Cd36, and Mcp6 was demonstrated in colonic tissue. The findings suggest the importance of the physiological functions of TSPO in ulcerative colitis and may serve as a basis for future studies aimed at dissecting the precise functions of this protein.

Cheng et al. used SPF C57BL/6J mice to investigate how high-altitude environments can damage colonic tissues. For this, animals were exposed to high altitude conditions (4,010 m), for 12, 24, or 48 h. The animals were euthanized, and the colonic damage was evaluated. High altitudes caused colonic inflammation and reduced luminal oxygen concentration. It also damaged the protective intestinal barrier and lead to increased expression of hypoxia-induced factors, including HIF-1α, NF-κB, and STAT1, and inflammatory factors, such as IFN-γ, IL-6, and TNF-α.

Finally, using a classic model of TNBS-induced ulcerative colitis, Dong et al. studied the role of the P2X3 receptor in cross-organ sensitization during colonic inflammation in rats. The contractility of the bladder detrusor muscle, as well as the micturition function, the intercontractile interval, and the maximum bladder pressure of the animals, were evaluated. Despite colonic inflammation, the bladder showed no histological alterations. However, there was an increase in the expression of the P2X3 receptor in the colonic tissues and dorsal root ganglion samples of colitis animals 3 days after the instillation of TNBS. Animals with ulcerative colitis exhibited bladder and detrusor overactivity; the last was reversed by P2X3 receptor inhibition. Based on these results, the authors suggested that inhibition of P2X3 activation is an interesting strategy to control bladder overactivity during colonic inflammation.

There is still much to be explored and discovered regarding gastrointestinal tract barrier damage and the development of inflammatory diseases. The progress and refinement of scientific knowledge are made with significant contributions, like the ones we saw here. We would like to thank all the editors, reviewers, authors, and co-authors who took the time to contribute to the published manuscripts.
